# Acute exacerbation of pulmonary toxoplasmosis during corticosteroid therapy for immune thrombocytopenia: A case report and literature review

**DOI:** 10.1097/MD.0000000000028430

**Published:** 2021-12-23

**Authors:** Koji Omori, Naoto Imoto, Kazumi Norose, Matsuyoshi Maeda, Kenji Hikosaka, Shingo Kurahashi

**Affiliations:** a Department of Gastroenterology, Toyohashi Municipal Hospital, Aichi, Japan; b Department of Hematology and Oncology, Toyohashi Municipal Hospital, Aichi, Japan; c Department of Infection and Host Defense, Graduate School of Medicine, Chiba University, Chiba, Japan; d Department of Pathology, Toyohashi Municipal Hospital, Aichi, Japan.

**Keywords:** acute respiratory distress syndrome, immune thrombocytopenia, pulmonary toxoplasmosis, trimethoprim–sulfamethoxazole prophylaxis

## Abstract

**Rationale:**

Pulmonary toxoplasmosis (PT) is an infectious disease that can be fatal if reactivation occurs in the recipients of hematopoietic stem cell transplantation (HSCT) who were previously infected with *Toxoplasma gondii*. However, whether the toxoplasmosis reactivation is an actual risk factor for patients receiving immunosuppressive therapies without HSCT remains unclear. Therefore, reactivated PT is not typically considered as a differential diagnosis for pneumonia other than in patients with HSCT or human immunodeficiency virus (HIV).

**Patient concerns:**

A 77-year-old man presented with fever and nonproductive cough for several days. He was hospitalized due to atypical pneumonia that worsened immediately despite antibiotic therapy. Before 4 months, he was diagnosed with immune thrombocytopenia (ITP) and received corticosteroid therapy. Trimethoprim–sulfamethoxazole (ST) was administered to prevent *pneumocystis* pneumonia resulting from corticosteroid therapy.

**Diagnosis:**

The serological and culture test results were negative for all pathogens except *T. gondii* immunoglobulin G antibody. Polymerase chain reaction, which can detect *T. gondii* from frozen bronchoalveolar lavage fluid, showed positive results. Therefore, he was diagnosed with PT.

**Intervention:**

ST, clindamycin, and azithromycin were administered. Pyrimethamine and sulfadiazine could not be administered because his general condition significantly worsened at the time of polymerase chain reaction (PCR) examination.

**Outcomes:**

The patient died of acute respiratory distress syndrome despite anti-*T. gondii* treatment. An autopsy revealed a severe organizing pneumonia and a small area of bronchopneumonia.

**Lessons:**

PT should be considered as a differential diagnosis in patients with pneumonia, particularly in seropositive patients who receive immunosuppressive therapies even for other than HSCT or HIV.

## 1 Introduction

Toxoplasmosis is caused by the protozoan parasite *Toxoplasma gondii.* Further, it is an opportunistic infection that can be fatal among immunocompromised patients such as those with human immunodeficiency virus (HIV) and those who received hematopoietic stem cell transplantation (HSCT).^[1–3]^ Pulmonary toxoplasmosis (PT) is not rare among HSCT recipients previously infected with *T. gondii*. The serological assessment of anti-*Toxoplasma* antibodies is recommended before starting immunosuppressive therapies.^[4]^ However, in patients treated with immunosuppressive drugs, PT is not commonly considered as a differential diagnosis for pneumonia, even though the condition is a common complication of *T. gondii* infection. There are few reports about reactivated PT without HIV infection or HSCT. However, whether the reactivation of toxoplasmosis is an actual risk among patients receiving immunosuppressive therapies remains unclear.

Herein, we present a patient who died of acute respiratory distress syndrome (ARDS) that was likely attributed to PT during corticosteroid therapy for immune thrombocytopenia (ITP). Moreover, a literature review of case reports about PT in patients without HIV infection or organ transplantation was performed.

## 2 Case report

A 77-year-old man presented to the hematologic department due to fever and nonproductive cough for several days. He visited the general internal medicine department to seek consultation for fever and colds approximately 4 months back. His blood test results showed thrombocytopenia (platelet count: 1000/μL). Thus, he was referred to the hematology department. He was then diagnosed with virus-associated or drug-induced ITP as bone marrow examination revealed the typical signs of ITP. Moreover, other differential diagnoses for thrombocytopenia such as collagen and aplastic diseases and hematological malignancies were ruled out. The patient was initially treated with prednisolone (PSL) 1 mg/kg (60 mg/day), and he had good response. During the current admission, the PSL dose was tapered to 10 mg/day. At the time of PSL therapy, trimethoprim–sulfamethoxazole (ST) 80/400 mg/day was administered as a prophylactic treatment for *pneumocystis* pneumonia. The patient had no comorbidities other than ITP. Further, he had no history of contact with animals (particularly cats), eating raw meat, or recent visits at certain areas considered at high risk for *T. gondii*. His medical or family history was unremarkable.

**Figure 1:**
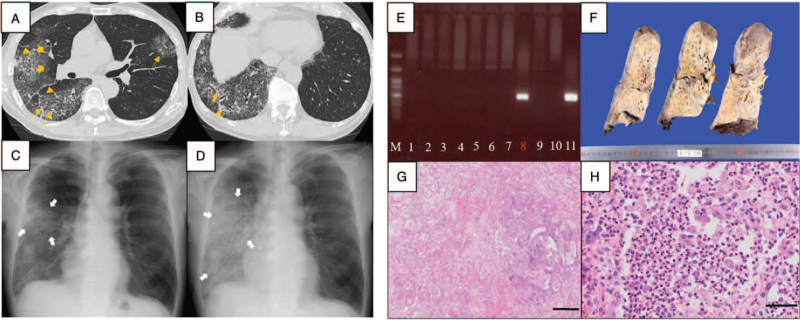
(A, B) Computed tomography findings during hospitalization. The right lung lobe mainly presented with areas with ground glass opacity (broad orange arrows), consolidation (thin orange arrow), and hypertrophy of the interlobular septa (orange triangles). (C, D) Plain chest radiography (C) during hospitalization and (D) at day 4. An infiltrative shadow (white arrows) in the right lobe spread immediately within 4 days. (E) Nested polymerase chain reaction results. Genomic DNA was purified from peripheral blood leukocytes (PBL) and bronchoalveolar lavage fluid (BALF). PCR was performed using primers specific for the *T. gondii B1* gene. Lane M: molecular size marker; lanes 1–6: PBL; lanes 7–9: BALF; lane 10: negative control; lane 11: positive control. *T. gondii* DNA was identified in lane 8. (F–H) Autopsy findings. (F) Macroscopic finding of the right lung. The lung had diffuse consolidation. (G) Microscopic finding revealed extensive organizing pneumonia with bronchopneumonia focus. Scale bar: 400 μm. (H) Infiltration of neutrophils and macrophages are observed in bronchopneumonia. Scale bar: 50 μm.

The vital signs (including oxygen saturation [96%] on room air and axial temperature) upon admission were normal. Coarse crackles were auscultated in the right lower lung field. Blood examination showed leukocytosis (white blood cell count: 16,390/μL, neutrophil ratio: 93%, and lymphocyte ratio: 2%). Blood chemistry revealed elevated C-reactive protein (27.51 mg/dL), lactate dehydrogenase (LDH) (277 U/L), and *β*-D glucan (20.0 pg/mL) levels. Computed tomography scan and plain chest radiography revealed a mixed appearance of ground glass opacity, consolidation, and hypertrophy of the interlobular septa in the whole right lobe and the left upper lobe (Figures 1A, 1B, and 1C). The patient was diagnosed with pneumonia and admitted to the hematology department.

**Figure 2:**
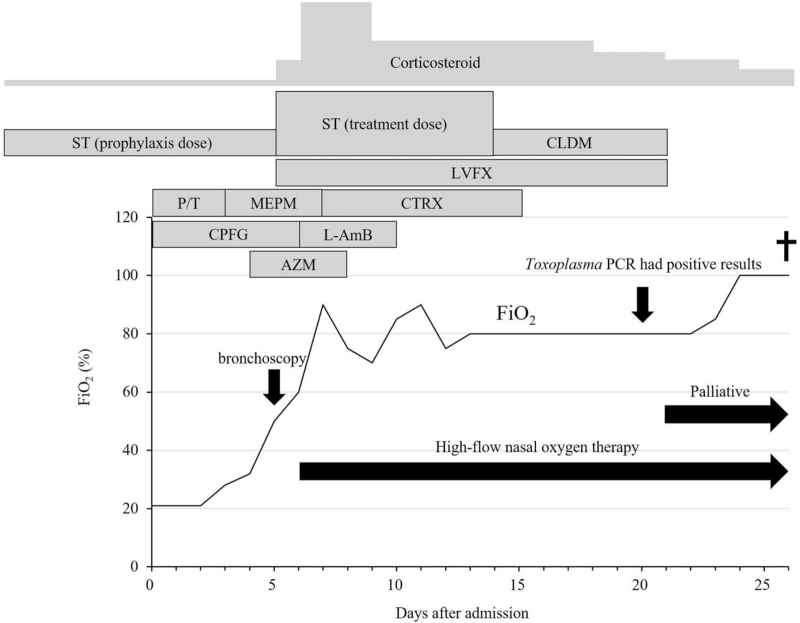
Clinical course of the patient. The FiO_2_ conversion before high-flow nasal oxygen was as follows: room air: 21%, oxygen flow rate of 2 L/min via nasal cannula: 28%, oxygen flow rate of 3 L/min via nasal cannula: 32%, oxygen flow rate of 6 L/min via a face mask: 50%. AZSM = azithromycin, CLDM = clindamycin, CPFG = caspofungin, CTRX = ceftriaxone, L-AmB = liposomal amphotericin B, LVFX = levofloxacin, MEPM = meropenem, P/T = piperacillin/tazobactam, ST = trimethoprim–sulfamethoxazole.

Piperacillin/tazobactam and caspofungin (CPFG) were administered as an empirical therapy. However, pneumonia worsened immediately (Fig. 1D). Hence, the treatment was changed to meropenem, azithromycin, and CPFG. After performing bronchoscopy on day 5 after admission, treatment with ST 960/4800 mg/day, PSL 40 mg/day, and levofloxacin were started for the management of *pneumocystis* pneumonia and other types of bacterial pneumonia. On day 6, the patient's respiratory status deteriorated, and it did not stabilize with oxygen therapy at 15 L/min via a reservoir mask. Blood chemistry revealed elevated LDH (368 U/L), Krebs von den Lungen-6 (1218 U/mL), pulmonary surfactant protein-D (1026 ng/mL), pulmonary surfactant protein-A (97.7 ng/mL), fibrin degradation product (251 μg/mL), and ferritin (1611 ng/mL) levels. Thus, the patient was diagnosed with ARDS and disseminated intravascular coagulation (DIC). Next, high-flow oxygen therapy via a nasal cannula was started in addition to corticosteroid pulse and DIC therapy. All examinations, which were performed to determine the etiology during the study period, had negative results. These included multiple sputum and blood cultures; bronchoalveolar lavage fluid (BALF) culture; Grocott staining of BALF; polymerase chain reaction (PCR), which can detect *Pneumocystis jirovecii* and *Legionella* from BALF and *Mycobacteria* from BALF and sputum; and testing for aspergillus antigen, HIV antibody, cytomegalovirus antigenemia, and *Chlamydia* antibody. The patient's *β*-D glucan level normalized on day 4. On day 7, as *Pneumocystis* was ruled out, the ST dose was reduced to 640/3200 mg/day. On day 10, the patient tested positive for anti-*Toxoplasma* immunoglobulin G antibody (38 IU/mL) and negative for anti-*Toxoplasma* IgM antibody. Moreover, his anti-*Toxoplasma* IgG avidity index was 0.54, thereby indicating chronic infection caused by *T. gondii*. To obtain a definitive diagnosis of *T. gondii* infection, we collected blood samples on day 13 and frozen BALF on day 5 for nested PCR.^[5]^ However, as there was a risk of CPFG refractory fungal infection, CPFG was changed to liposomal amphotericin B. In addition, as PT or anaerobic pneumonia was suspected, clindamycin was added to the therapy. His general status might be temporarily reasonable, and his FiO_2_ ratio did not decrease below 60%. On day 14, as the patient had difficulties in taking ST and his platelet count decreased, ST was discontinued. On day 20, nested PCR revealed the presence of *Toxoplasma* in the BALF, but not in the blood (Fig. 1E). As his general condition further deteriorated and both lungs had severe infiltration, treatment with pyrimethamine and sulfadiazine, which are the mainstay therapy for toxoplasmosis, could not be started. Hence, the patient and his family were consulted regarding palliative treatment. The patient died from respiratory failure on day 26. Figure 2 shows the clinical course.

On autopsy, the lungs were heavy (left: 950 g and right: 850 g). The cut surface showed congestion and extensive tan-yellow consolidation (Fig. 1F). Histologically, there was organizing fibrosis in the alveolar spaces without remodeling of the lung architecture, thereby showing a pattern of organizing pneumonia and a small area of bronchopneumonia (Fig. 1G). Neutrophils and swollen macrophages infiltrated the bronchiole and alveolar spaces in the bronchopneumonia region (Fig. 1H). *Toxoplasma* was not apparent in these specimens, and PCR testing for *T. gondii* in the lung slides obtained during autopsy yielded negative results (data not shown). *Stenotrophomonas maltophilia* (1+) was identified via culture of the lung autopsy specimen. However, it was not apparent under microscopic view during autopsy.

## 3 Discussion

Our patient who was treated with glucocorticoid therapy for ITP died of ARDS, and the only causative pathogens even based on the pathological autopsy were *S. maltophilia* and *T. gondii*. *S. maltophilia* can be detected in routine culture tests. However, in the current case, pneumonia could not be attributed to *S. maltophilia* as it was not detected in any tests other than the lung culture by autopsy. Multiple sputum, blood, and BALF cultures tested negative for *S. maltophilia*. In addition, its presence was not validated via pathological investigation. *S. maltophilia* might have colonized in the lungs, which was caused by microbial substitution due to the continuous use of broad-spectrum antimicrobials for approximately 1 month while the patient's general condition was poor. In the current case, PT was the most likely diagnosis due to the following reasons: First, PCR of BALF showed positivity to *T. gondii*, which strongly indicated that the lungs were infected with *T. gondii*. Second, the tests for the other causative of microorganisms of pneumonia, except for *S. maltophilia*, yielded negative results. Third, the clinical and pathological findings were consistent with those described in previous reports of PT in HSCT recipients.^[4]^ In addition, *T. gondii* could have easily reactivated in our patient as his immune status deteriorated due to treatment with glucocorticoids. *T. gondii* and its DNA were not found in the autopsy slides, probably because the parasites had been successfully eradicated via treatment with ST, clindamycin, and azithromycin at therapeutic doses for more than 1 week. However, the patient died of concomitant ARDS and DIC that could not be controlled.

Commonly, PT is only mildly symptomatic in immunocompetent hosts. However, it can be life-threatening in significantly immunocompromised hosts.^[6]^ The symptoms of PT are nonspecific, and these include fever, nonproductive cough, myalgia, arthralgia, lymphadenopathy, and dyspnea. Moreover, the laboratory findings are nonspecific, which include lymphopenia, thrombocytopenia, rhabdomyolysis, and LDH elevation.^[7]^ Further, an elevated C-reactive protein level is nonspecific and may be influenced by differences in the underlying disease, treatment, and/or causative *T. gondii* strain.^[4,8,9]^ The diagnosis of PT can become more challenging due to computed tomography findings such as ground glass opacity and peribronchovascular thickening, which indicate other pulmonary conditions of atypical pneumonia.^[4,6]^ This disease might be misdiagnosed, or proper treatment could be delayed because of its nonspecific clinical features and imaging findings and insufficient knowledge among clinicians. Thus, it has a high mortality rate.^[4,10,11]^

There are no systematic reports about toxoplasmosis in immunocompromised patients, except HSCT recipients and patients with HIV. There are several case reports of toxoplasmosis. However, most discussed about cerebral toxoplasmosis.^[12,13]^ A PubMed search was performed on August 8, 2021 using the phrase PT, and 932 articles were identified. Nevertheless, only 8 reports were considered after the exclusion of studies with transplant recipients, patients with HIV, disseminated cases with main symptoms other than respiratory, nonhuman cases, articles published in languages other than English, and unknown cases (Table 1).^[8,9,11,14–18]^ As shown in Table 1, in most cases, the patient's condition improved with *T. gondii-*specific approaches such as *T. gondii* serologic testing and pyrimethamine–sulfadiazine treatment despite the presence of various and nonspecific underlying diseases and symptoms. Thus, suspected *T. gondii* and availability of pyrimethamine–sulfadiazine are important for the timely initiation of appropriate treatment.

**Table 1:** Case reports of pulmonary toxoplasmosis in patients without HIV infection or organ transplantation.

Authors^∗^	Sex	Age	Underlying disease	Treatment at toxoplasmosis onset	Symptoms	Affected organs	Diagnostic method	Primary infection or reactivation^∗∗^	Toxoplasmosis treatment	Outcome
Brown et al^[14]^	F	41	Acute myelomonocytic leukemia	Consolidation chemotherapy	Cough, dyspnea	Lung, spleen, bone marrow, lymph node, pancreas	Autopsy	Reactivation	None	Death
De Salvador-Guillouët et al^[8]^	M	19	None	None	Fever, fatigue, dyspnea	Lung	Histology (BALF), serology (IgG, IgM, and IgG avidity)	Primary	Pyrimethamine and sulfadiazine	Improved
Leal et al^[15]^	M	41	None	None	Fever, myalgia, headache, nausea, vomiting	Lung, cerebrospinal fluid	Serology (IgG and IgM), PCR (cerebrospinal fluid)	Primary	Pyrimethamine and sulfadiazine	Improved
de Souza Giassi et al^[9]^	M	36	Type 2 diabetes	None	Fever, cough, dyspnea	Lung	Serology (IgG, IgM, and IgG avidity)	Primary	Pyrimethamine and sulfadiazine	Improved
	F	56	Type 2 diabetes							
	F	38	None							
Lu et al^[16]^	F	64	Lung cancer	None	Nonproductive cough, chest pain	Lung	Histology (BALF), serology (IgG and IgM)	Primary	Unknown	Unknown
Abdulkareem et al^[11]^	F	55	Inflammatory arthritis	Methotrexate and corticosteroids	Nonproductive cough, shortness of breath	Lung	Histology (lung biopsy)	Reactivation	Trimethoprim-sulfamethoxazole	Improved
Matsuzawa et al^[17]^	M	74	Myelodysplastic syndrome	None	Fever, dyspnea	Lung	Serology (IgG and IgM)	Primary	Pyrimethamine and sulfadiazine	Improved
Steinhauser Motta et al^[18]^	M	30	None	None	Dry cough, dyspnea, cervical lymphadenopathy	Lung, lymph node, liver, spleen	Serology (IgG and IgM), PCR (BALF)	Primary	Pyrimethamine and sulfadiazine	Improved
Omori et al, (current report)	M	77	Immune throbocytopenia	Corticosteroids and trimethoprim-sulfamethoxazole	Fever, nonproductive cough	Lung	PCR (BALF)	Reactivation	Trimethoprim-sulfamethoxazole and clindamycin^∗∗∗^	Death

∗ Reference number.

∗∗ *Toxoplasma gondii* IgM-positive cases were defined as primary.

∗∗∗ These medicines were used as empiric therapy targeting not only *Toxoplasma* pneumonia but also *Pneumocystis* and anaerobic pneumonia. BALF = broncheoalveolar lavage fluid, PCR = polymerase chain reaction.

The measurement of anti-*Toxoplasma* IgG antibody is recommended before HSCT to reduce the incidence of severe infection.^[2]^ However, this is not generally performed on patients receiving immunosuppressive therapies, as the reactivation risk associated with other immunosuppressive therapies such as corticosteroid administration and chemotherapy is not elucidated. In addition, the diagnosis of PT requires considerable effort and time in Japan where *Toxoplasma* PCR cannot be routinely performed despite the large number of target patients. Thus, it is challenging to differentiate PT in *Toxoplasma-*seropositive patients compared with HSCT recipients in Japan. Furthermore, patients receiving immunosuppressive therapies (such as corticosteroids and chemotherapy) have a milder level of immunosuppression than HSCT recipients. Therefore, the positivity rate of *Toxoplasma* PCR can be significantly lower in patients receiving immunosuppressive therapies than in HSCT recipients. To overcome these issues, *Toxoplasma*-seropositive patients with an unknown cause of pneumonia should directly undergo transbronchial lung biopsy via bronchoscopy and BALF cytology and pathologists should cautiously investigate for the presence of *Toxoplasma*. PT might be overlooked because of its rarity. Hence, clinicians must be aware of which patients should be suspected with PT and how to investigate for toxoplasmosis.

Toxoplasmosis can be effectively prevented with ST. That is, ST at a dosage of 80/400 mg/day can be administered daily. However, ST at a dose of 160/800 mg/day is generally administered either twice or 3 times a week. However, previous reports have documented the onset of toxoplasmosis during prophylactic ST treatment (80/400 mg/day),^[19]^ as in the current case. Failed prophylactic treatment can be explained by inefficient drug absorption, intolerance, and discontinuation.^[20]^ ST prophylactic regimens comprising ST administration less than 3 times a week have a lower efficacy than regimens requiring more frequent administrations.^[19]^ In addition, anti-*T. gondii* medications (both chemoprophylaxis and treatment for *T. gondii*) are not effective against latent bradyzoite forms, such as cysts, involved in chronic infection, and they do not prevent encysted-bradyzoite reactivation.^[20]^ Thus, clinicians should be aware that toxoplasmosis can still occur in patients who have received prophylactic ST therapy.

The current case showed that pneumonia caused by *T. gondii* reactivation can occur during corticosteroid and prophylactic ST therapies. PT is challenging to diagnose because of its nonspecific clinical features and laboratory and imaging findings. However, it should be considered as a differential diagnosis for pneumonia, particularly in seropositive patients receiving immunosuppressive treatments.

## Acknowledgments

We want to thank all the staffs involved in medical treatment. We also would like to thank Enago (www.enago.jp) for English language review.

## Author contributions

**Conceptualization:** Naoto Imoto.

**Data curation:** Koji Omori, Naoto Imoto.

**Formal analysis:** Naoto Imoto.

**Investigation:** Kazumi Norose, Matsuyoshi Maeda, Kenji Hikosaka.

**Methodology:** Naoto Imoto.

**Project administration:** Naoto Imoto.

**Supervision:** Shingo Kurahashi.

**Validation:** Kazumi Norose, Matsuyoshi Maeda, Kenji Hikosaka, Shingo Kurahashi.

**Visualization:** Naoto Imoto, Kazumi Norose.

**Writing – original draft:** Koji Omori, Naoto Imoto.

**Writing – review & editing:** Kazumi Norose.
